# The Emergence of New-Onset Obsessive and Compulsive Disorder in an Adolescent During COVID-19 Pandemic

**DOI:** 10.7759/cureus.17907

**Published:** 2021-09-12

**Authors:** Albulena Sejdiu, Sayeda A Basith, Victoria Ayala, Subani Maheshwari

**Affiliations:** 1 Psychiatry, St.Cyril and Methodius University, Skopje, MKD; 2 Psychiatry and Behavioral Sciences, Medical University of the Americas, Charlestown, KNA; 3 Psychiatry, Ross University School of Medicine, Bridgetown, BRB; 4 Psychiatry, Christiana Care, Wilmington, USA

**Keywords:** obsessive compulsive disorder, ybocs, clomipramine, olfactory, child and youth mental health, covid 19

## Abstract

The COVID-19 pandemic has had a huge toll on both the physical and mental health of people around the globe. Neuropsychiatric symptoms, as well as long-term sequelae, have been demonstrated in those afflicted with COVID-19. These symptoms range from cognitive, attention deficit, new-onset anxiety, depression, psychosis, seizures, and post-traumatic stress. Prolonged lockdown led to social isolation which negatively affected the mental well-being of many individuals. This particularly caused a relapse of psychiatric symptoms due to stress related to the COVID-19 pandemic. It sparked an increase in hoarding behaviors such as obtaining germicidal and cleaning supplies. In this report, we present a case of an adolescent male presenting with a new onset of obsessive-compulsive disorder with symptoms similar to olfactory hallucinations and olfactory reference syndrome in the setting of the COVID-19 pandemic.

## Introduction

The COVID-19 pandemic has affected over 200 million people worldwide. It has been noted that these numbers underestimate the burden of COVID-19, as only a small portion of acute infections have been diagnosed and reported [1].

Neuropsychiatric symptoms, as well as long-term sequelae, have been demonstrated in those afflicted with COVID-19. These symptoms range from cognitive, new-onset anxiety, depression, psychosis, seizures, and post-traumatic stress. Neuropsychiatric symptoms could be a consequence of micro strokes and neuronal damage, and symptoms vary based on which area of the brain is involved [2]. The incidence of neuropsychiatric symptoms is higher amongst patients who have been hospitalized and are more prominent in those who were admitted to the intensive care unit or have experienced encephalopathy [3].

Prolonged lockdown led to social isolation which negatively affected the mental well-being of many individuals [4]. This particularly caused a relapse of psychiatric symptoms due to stress related to the COVID-19 pandemic. It sparked an increase in behaviors such as stockpiling germicidal and cleaning supplies, more than what can be used, due to the fear of limited stocks of cleaning supplies being available during the early phase of the pandemic. Most commonly, obsessions of contamination and compulsive behavior of handwashing have been seen [4]. The use of masks has led to worsening symptoms of certain pre-existing mental health conditions in some individuals such as paranoia and anxiety [5].

In this report, we present a case of an adolescent male presenting with a new onset of obsessive-compulsive disorder with symptoms similar to olfactory hallucinations and olfactory reference syndrome in the setting of the COVID-19 pandemic.

## Case presentation

A 14-year-old white male with a history of high functioning autism and attention-deficit/hyperactivity disorder (ADHD) and no past medical history presented to the emergency department with worsening anxiety, and new-onset compulsions with cleaning, and olfactory hallucinations. Upon interview, the patient vaguely correlated the onset of these symptoms to the start of the COVID-19 pandemic crisis. The patient was not diagnosed with COVID-19 infection previously. His above-mentioned symptoms had heightened in the past two weeks. He reported that he was focused on avoiding touching the door handles at home and had been washing his hands multiple times throughout the day. The patient and his parents noted that the patient's hands were getting chapped. He also admitted to sniffing cleaning supplies multiple times a week and rubbing it on his skin and around his nose and face. In addition, he reported intermittent episodes of smelling a foul odor at home and church, and inside the vehicle for the past two months. He could not further elaborate on the smell but expressed that the odor made him feel annoyed. He reported increased irritability and poor frustration tolerance especially when he was around his sister and father. Their fetid smell/odor further distressed the patient with thoughts about his hygiene, resulting in crying episodes if he did not quickly find the cleaning supplies. He endorsed a nonspecific history of depressed mood and anhedonia in which he no longer enjoyed his favorite television shows for the past few months.

The patient refused to touch or use items previously handled by his sister or father and screamed if either one was in the same room as him. One month prior, the patient was prescribed asenapine 5mg/day by his pediatrician for suspected olfactory hallucinations; nevertheless, he was noncompliant, as the medication was found stashed in his bedroom vent. Any attempt to give the patient his recommended dose resulted in aggression as per the parents. No other medication trials were noted in the past, and baseline laboratory workup including complete blood count, comprehensive metabolic panel, thyroid panel, urine drug screen, and EKG were within normal limits. A consult was placed for the medical team to consider ordering MRI and baseline EEG but it was ruled out as there were no other concerning neurological findings on examination and the likelihood of a neurologic explanation for his symptoms were considered low, based on his presentation. Furthermore, on obtaining a detailed psychiatric history it became increasingly evident that the patient's preoccupation with putrid smells was accompanied by compulsive behaviors such as rubbing cleaning supplies on his hands and around his nose, which led to relief in the patient's distress. The patient had no other medical or surgical history. It is to be noted that the patient had high functioning autism with no previous struggles in maintaining activities of daily living and was getting average grades at school. There were no previously mentioned preoccupations with cleaning behaviors or accumulating cleaning supplies, that were noted by the parents prior to this episode. The patient had baseline stereotypical interests pertaining to sports and in particular following NFL teams. Both he and his family did not report a previous history of specific obsessions of cleaning or following ritualistic behaviors in the past. No previous history of tics, mannerisms, tremors was reported. There was no family history of psychiatric or neurological illnesses. 

At the time of admission, the patient denied passive or active suicidal and homicidal ideations, and/or visual and auditory hallucinations. On detailed evaluation, it was found that the patient had olfactory obsessions and not olfactory hallucinations as the patient had some insight into his symptoms which were ego dystonic in nature. He also did not meet the criteria for olfactory reference syndrome as it entails distress from smell coming from self and not others. Treatment options were discussed with his parents including selective serotonin reuptake inhibitors (SSRIs) and tricyclic anti-depressants. Given the parental concern about SSRIs leading to an adverse effect in one of their close friends, they preferred a trial of a tricyclic anti-depressant, namely clomipramine. The parents were educated about the potential side effects form tricyclic anti-depressants at length. Baseline EKG was obtained and was noted to be a normal study.The patient was started on clomipramine 25mg/day, which was titrated up to 100mg/day by the time of discharge. Repeat EKG in the partial hospitalization program was also noted to be within normal limits. The children’s Yale-Brown obsessive-compulsive scale score changed from 23 at admission to 15 at discharge within the three-week inpatient treatment. The patient reported significant reduction in his distress related to putrid smells as well as anxiety overall. It was also noted that the patient was initially wearing long sleeve shirts to avoid touching doorknobs, which gradually resolved towards the end of his hospital stay. He developed good insight into his symptoms and used coping skills such as relaxation and deep breathing to address his anxiety symptoms. He was discharged to his parents' home with follow-up at a partial program and referral for therapy geared towards exposure and response prevention. The patient was successfully able to complete his treatment at the partial program and transfer to an outpatient clinic.

## Discussion

The COVID-19 pandemic has affected children and adults with obsessive-compulsive disorder (OCD) especially due to constant warnings on social media and TV about maintaining hygienic precautions which have in some cases induced an exacerbation of compulsive symptoms of handwashing and excessive cleaning [6]. It has exacerbated the frequency of cleaning compulsions and obsessional fear of contamination in many patients during the pandemic [7, 8]. The other factor that may be a trigger for exacerbating OCD symptoms is potential trauma from the fear of contracting COVID-19 and the fear of seeing and hearing about serious illnesses and deaths occurring during this pandemic on media [9]. The psychosocial impact of the COVID-19 pandemic has led to the worsening of depressive and anxiety disorders including panic attacks, illness anxiety, mass hysteria, and OCD, and this has been mostly neglected [4].

During the COVID-19 pandemic, patients with psychiatric illnesses have faced certain obstacles because of the nature of the psychiatric treatments, which require close contact, social interactions, and group and individual therapies [10]. Fortunately, digital technologies enabling social interactions and therapy sessions have been effective in psychiatric patient management [11]. So, patients who have more intense reactions related to the COVID-19 pandemic and receive virtual psychotherapeutic treatment during the pandemic can help reduce their OCD symptoms [12]. It is also important to provide collaborative care for both the physical and mental health needs of patients during the ongoing pandemic. Families and healthcare providers should closely monitor high-risk patients and provide support to improve their overall emotional well-being [13].

Since there are very few approved treatments available for treating hospitalized COVID-19 patients, there has been an increase in the use of off-label medications for the same. Clinicians should be aware of potential drug-drug interactions between these therapies and psychotropic medications commonly used in inpatient treatment across the ages [14-16]. Clomipramine is the only tricyclic anti-depressant (TCA) approved by the food and drug administration (FDA) for treating OCD in ages 10 and older, with the potent ability to inhibit serotonin and norepinephrine reuptake and dopamine-blocking effects [17,18]. As per a meta-analysis study, clomipramine was more effective than sertraline, fluoxetine, and fluvoxamine for the treatment of OCD [18].

It is noteworthy that SSRIs are the first line of treatment for pediatric OCD. However, in this particular case, TCA was started because of a strong parental preference for a non-SSRI option. Baseline and repeat EKGs obtained were both noted to be normal studies. This case report emphasizes the impact that the COVID-19 pandemic has had on the mental health of teenagers in the backdrop of stressors from the social isolation that has led to the new onset of psychiatric co-morbidities. While there are studies on the impact of COVID-19 on existing OCD symptoms in pediatric patients [12], this case report is one of the few studies if any (we did not find any other similar case reports in pediatrics), with new-onset OCD in a child in the context of stressors surrounding increased social isolation and worries about maintaining proper hygiene during the COVID- 19 pandemic. It is also interesting that the initial presentation focused on olfactory hallucinations, but on getting a detailed history, it was found to be a symptom of OCD and not psychosis or secondary to a neurological process, which responded well to a trial of TCA medication. This case further emphasizes the need for clinicians to get a more detailed history especially in the current times of the surrounding stressors and worries related to the pandemic. Increased access to mental health appointments for medication management as well as therapy via telepsychiatry has been very helpful in these challenging times [19].

## Conclusions

The COVID-19 pandemic has had a huge toll on both the physical and mental health of people around the globe. Mental health co-morbidities, especially in the pediatric age group, have had a huge impact because of increased social isolations as well as challenges surrounding decreased mental health appointments secondary to social distancing measures. It's prudent for clinicians to pay close attention to mental health screening measures in the patients that they are seeing and make appropriate referrals as needed.
